# Impact of additional theoretical training program in the diagnosis of the fetal head position during labor: a prospective observational study

**DOI:** 10.1186/s12884-023-05472-1

**Published:** 2023-03-07

**Authors:** Ye Song, Yun Qu, Jia Liu, Hongjing Jia, Yongfei Yue, Xuepiao Zhao

**Affiliations:** 1 Department of Obstetrics and Gynecology, The Affiliated Suzhou Hospital of Nanjing Medical University, Suzhou Municipal Hospital, No. 26 Daoqian Street, Gusu District, 215002 Suzhou, Jiangsu China; 2 Department of Medical Ultrasound, The Affiliated Suzhou Hospital of Nanjing Medical University, Suzhou Municipal Hospital, Suzhou, Jiangsu China; 3 Department of Obstetrics and Gynecology, The Affiliated SuQian first people’s Hospital of Nanjing Medical University, SuQian, Jiangsu China

**Keywords:** Labour, Occiput posterior position, Fetal head position, Transvaginal digital examination

## Abstract

**Background:**

The accuracy of transvaginal digital examination in determining foetal head position is not high enough. This study aimed to evaluate whether an additional training on our new theory could improve the diagnostic accuracy of the foetal head position.

**Methods:**

This was a prospective study conducted at a 3a grade hospital. The study included 2 residents in their first year of training in obstetrics without prior experience in transvaginal digital examination. In the observational study, 600 pregnant women without contraindications to vaginal delivery were included. Two residents were simultaneously trained in the theory of traditional vaginal examination, but resident B received an additional theoretical training program. The pregnant women were randomly assigned to have the foetal head position examined by resident A and resident B. The foetal head position was then confirmed by ultrasound, which was performed by the main investigator. After 300 examinations were independently performed by each resident, the accuracy of foetal head position and perinatal outcomes were compared between the two groups.

**Results:**

During the 3-month period, 300 post training transvaginal digital examinations were performed by each resident in our hospital. The two groups were found to be homogeneous for age at delivery, BMI before delivery, parity, gestational weeks at delivery, the rate of epidural analgesia, foetal head position, presence of caput succedaneum, presence of moulding and foetal head station(*p* > 0.05). The diagnostic accuracy of head position by digital examination was higher for resident B, who was subjected to an additional theoretical training program, than for resident A (75.00% vs. 60.67%, *p* < 0.001). There were no significant differences in maternal and neonatal outcomes between the two groups (*p* > 0.05).

**Conclusion:**

An additional theoretical training program for residents increased the accuracy of vaginal assessment of foetal head position.

**Trial registration:**

Registered at Chinese Clinical Trial Registry Platform (ChiCTR2200064783), October 17, 2022. https://www.chictr.org.cn/edit.aspx?pid=182857&htm=4

## Background

Foetal head malposition is closely related to vaginal delivery failure and maternal-foetal morbidity during operative vaginal delivery [1], and a persistent occipital posterior position may lead to prolonged labour or even caesarean Sect. [2]. The incidence of persistent posterior occipital position is approximately 5–12% [3]. If persistent occipital posterior position is not handled in time, there may be an increased risk of prolonged labour, perineal lacerations, and neonatal injuries. Many of these risks could have been avoided if the occipital posterior position had changed to the occipital anterior position. Foetal head position is an important factor affecting vaginal delivery, and determining foetal position by transvaginal digital examination is the premise of manual rotation of the foetal occiput (MRFO). Accurate diagnosis of the foetal head position is a prerequisite for instrumental vaginal delivery (the vacuum cup or forceps). Therefore, a practical method for identifying foetal head position is useful in assessing the progress of labour.

Ultrasonic diagnosis of the foetal head position is more accurate than transvaginal digital examination [4, 5]. Previous studies have reported that the accuracy of transvaginal digital examination in determining foetal head position range from 24 to 95% at different hospitals [6–8]. However, the level of medical care is not consistent in different regions of the world, and not every maternity ward is equipped with ultrasound equipment and a doctor who can operate the ultrasound equipment. In emergency situations, transvaginal digital examination is a faster and more effective way to determine foetal head position than ultrasound. Determining foetal head position through vaginal examination requires a number of clinical practices that residents lack. Simulation-based training can improve the accuracy of digital vaginal examination to some extent [9]. The method of transvaginal digital examination of foetal head position in textbooks is complicated, and different physicians have different understandings of the method. New residents need a long time to accumulate experience to accurately judge foetal head position in clinical practice, it is necessary to develop a teaching method for residents to quickly master the determination of foetal head position.

Traditional methods require the identification of the anterior and posterior fontanelles to determine foetal head position, which can be difficult for residents. Our research team proposed a simplified method for determining foetal head position that may help residents master this skill in a relatively short time. We consider the frontal suture to be the key to distinguishing the anterior and posterior fontanelles. Therefore, we performed a pilot comparison study to examine the effects of the new teaching method in improving the diagnostic accuracy of the foetal head position.

## Materials and methods

This prospective study was approved by the Ethics Review Board of the affiliated Suzhou Hospital of Nanjing Medical University (K-2022-100-H01), and informed consent was signed by pregnant women. We have a prospective registration for our study on the Chinese Clinical Trial Registry Platform (Registration number: ChiCTR2200064783, Registration date: 17/10/2022). Pregnant women without obstetric and other complications who were awaiting childbirth in the delivery room of Suzhou Municipal Hospital between October 2022 and December 2022 were included in our study. The inclusion criteria were as follows: (1) age ≤ 35 years; (2) singleton gestation ≥ 37 weeks; (3) cervical dilation ≥ 5 cm; (4) rupture of membranes; and (5) no previous history of cervical surgery.

**Fig. 1:**
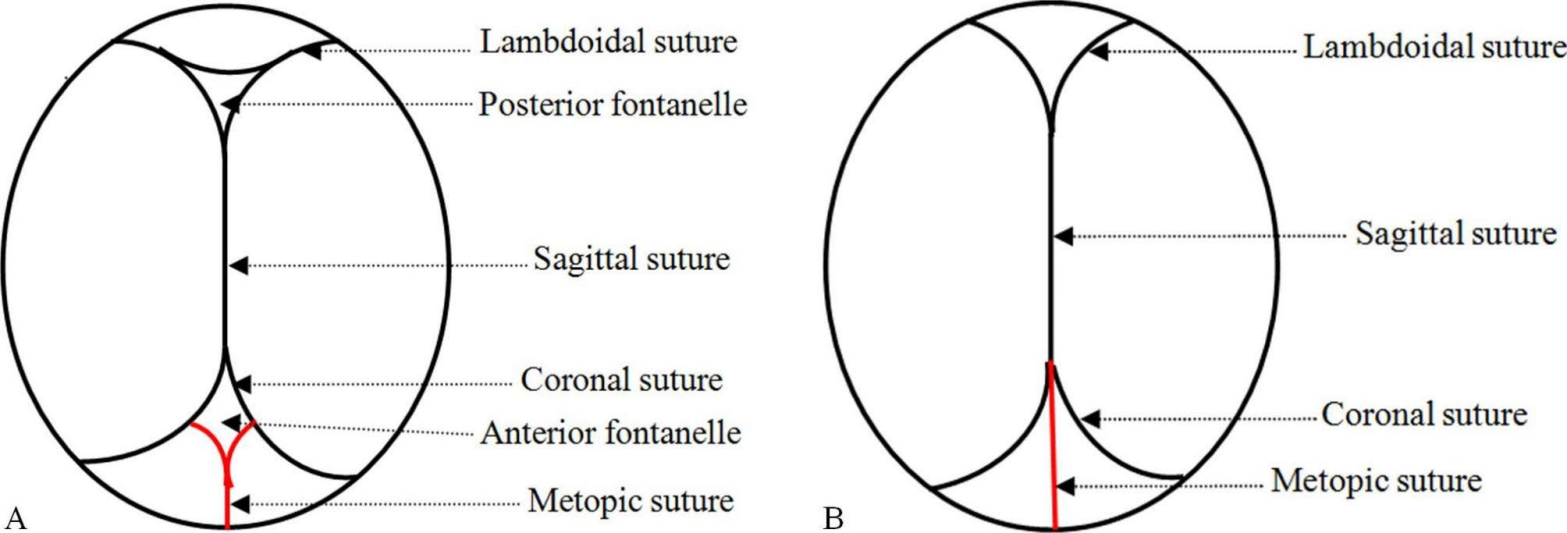
**A** Normal foetal head at term showing fontanels and sutures. **B** The fontanels and sutures of the foetal head compressed by the birth canal during labour

A sample size of 600 pregnant women was calculated, considering an improvement in agreement from 60 to 75%, and a power of 90%. Two obstetrics and gynecology residents participated in the study in their first year of residency. Prior to the study, two residents (Residents A and B) participated in a traditional 60-minute theoretical and practical teaching on determining foetal head position [10]. Resident B received an additional 30-minute of training on our new theory of determining foetal head position. This training is an important and correct way to guide residents to examine the position of the occiput. The anterior fontanelle is rhomboid and the posterior fontanelle is triangular (Fig. 1A). During labour, the foetal head is compressed by the birth canal, the foetal head is deformed and the bone sutures overlap, then the anterior fontanelle disappears in a diamond shape. Both the anterior and posterior fontanelles are triangular, but we can touch metopic sutures by digital examination in the triangle of the anterior fontanelle (Fig. 1B). Therefore, the metopic suture of the anterior fontanelle is the key suture that distinguishes the anterior fontanelle from the posterior fontanelle during labour (extra theoretical key point received by Resident B). The anterior fontanelle can be touched simultaneously with three sutures, while the posterior fontanelle can only be touched with two sutures.

**Fig. 2:**
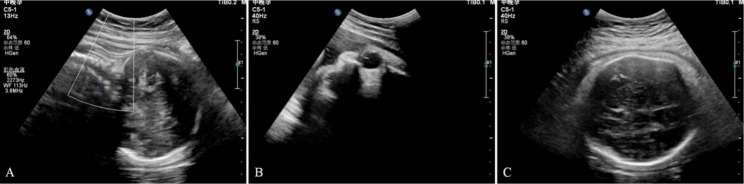
**A** Transabdominal ultrasound image of the foetal spine to determine the foetal head position. **B** Transabdominal ultrasound image of foetal orbits to determine foetal head position. **C** Transabdominal ultrasound image of foetal intracranial structures to determine foetal head position

After the digital examination, a sonographer immediately performed a transabdominal ultrasound (Affiniti 70, Philips, Holland) to determine foetal head position (Fig. 2). The position of the foetus was determined by the location of the foetal spine, orbits and midline intracranial structures [11]. Both residents were informed of the position of the foetal head on ultrasound. A senior obstetrician-gynecologist will assists the residents in managing labour if the women required MRFO or operative deliveries (vacuum, forceps or caesarean section). If the foetal heart is category III or labour progress is abnormal, we can try to rotate the foetal head by hand to be good for intrauterine resuscitation of the fetus and labour progress. Within five minutes after neonatal delivery, umbilical cord arterial blood was extracted for bedside blood gas analysis (i-STAT300G, Abbott Laboratories, America).

### Data analysis and statistics

Data are expressed as the mean standard deviation (SD) or as a number (percentage). Categorical variables between groups were tested using the chi-square or Fisher exact test. Continuous variables were compared between the two groups using the t test or Mann Whitney nonparametric equivalent test. All statistical analyses were performed by using SPSS 23.0 statistics software (SPSS, Inc., Chicago, IL, USA). A *p* value < 0.05 was considered statistically significant.

## Results

### Clinical characteristics of the patients

A total of 600 pregnancies screened randomly completed the trial. The women were divided into group A which was examined by resident A, and group B which was examined by resident B, with 300 women in each group. The two groups were found to be homogeneous regarding age at delivery, BMI before delivery, parity, gestational weeks at delivery and the rate of epidural analgesia (*p* > 0.05). There were no significant differences in foetal head position, presence of caput succedaneum, presence of moulding and foetal head station between the two groups (*p* > 0.05) (Table 1).

**Table 1:** Demographic characteristics of the studied groups

Parameter	Group A (n = 300)	Group B (n = 300)	*t/χ* ^*2*^	*p*-value
Age at delivery(years)	29.08 ± 3.78	28.68 ± 3.87	1.27	0.205
BMI before delivery (kg/m^2^)	25.90 ± 3.45	26.08 ± 3.61	0.632	0.528
Parity			0.52	0.471
Primipara, n (%)	245(81.67)	238(79.33)		
Multipara, n (%)	55(18.33)	62(20.67)		
Gestational weeks at delivery (week)	39.07 ± 1.16	38.95 ± 1.13	1.35	0.176
Epidural Analgesia, n(%)	223(74.33)	242(80.67)	3.45	0.063
Foetal head position assessed by ultrasound, n(%)			0.86	0.649
Occipitoanterior position	231(77.00)	223(74.33)		
Occipitoposterior position	31(10.33)	38(12.67)		
Occipitotransverse position	38(12.67)	39(13.00)		
Presence of caput succedaneum	17(5.67)	20(6.67)	0.26	0.611
Presence of moulding	9(3.00)	11(3.67)	0.21	0.649
Foetal head station			0.78	0.676
Above ischial spines	24(8.00)	29(9.67)		
At ischial spines	91(30.33)	95(31.67)		
Below ischial spines	185(61.67)	176(58.67)		

### Maternal and foetal perinatal outcome

There were no significant differences in the first stage of labour, second stage of labour and cervical dilation between exam and delivery between the two groups (*p* > 0.05). However, the diagnostic accuracy of head position by digital examination was higher for B resident compared with A resident subjected to additional training in our new method (75.00% vs. 60.67%, *p* < 0.001), which was assessed by means of ultrasound examination. There was no difference in exam to delivery interval, cervical dilation between exam to delivery, mode of delivery, volume of blood loss at 2 h after delivery and perineal outcome between the two groups (*p* > 0.05). Groups were similar in terms of birth weight, neonatal asphyxia rate and NICU admission (*p* > 0.05) (Table 2).

**Table 2:** Comparison of delivery outcomes between the studied groups

Parameter	Group A(n = 300)	Group B(n = 300)	*t/χ* ^*2*^	*p*-value
Duration of labour				
First stage of labour (minutes)	422.97 ± 116.60	411.64 ± 113.56	1.21	0.228
Second stage of labour (minutes)	54.18 ± 17.76	52.19 ± 11.23	1.64	0.101
Diagnostic accuracy by digital examination, n(%)	182(60.67)	225(75.00)	14.12	< 0.001
Exam to delivery interval (min)	91.42 ± 15.33	89.87 ± 17.99	1.13	0.257
Cervical dilation between exam and delivery (cm)	3.59 ± 0.97	3.65 ± 0.98	0.84	0.403
Mode of delivery, n(%)			0.72	0.697
Spontaneous deliveries	246(82.00)	253(84.33)		
Operative deliveries (vacuum or forceps)	21(7.00)	20(6.67)		
Caesarean section	33(11.00)	27(9.00)		
Volume of blood loss at 2 h after delivery (ml)	342.40 ± 140.30	334.96 ± 116.20	0.71	0.480
Perineal outcome, n(%)			0.85	0.653
I° perineal laceration	243(81.00)	251(83.67)		
II° perineal laceration	12(4.00)	9(3.00)		
Episiotomy	45(15.00)	40(13.33)		
Birth Weight(gram)	3339.13 ± 412.39	3330.68 ± 419.46	0.25	0.804
Umbilical artery PH	7.21 ± 0.08	7.22 ± 0.08	1.18	0.237
Neonatal asphyxia rate, n(%)	4(1.33)	5(1.67)	0.11	0.737
NICU admission	8(2.67)	10(3.33)	0.23	0.632

## Discussion

The foetal head position plays an important role during vaginal delivery and is one of the known predictors of labour outcome. Earlier studies by Shetty et al. [12] had observed that the detection of occiput posterior position by transvaginal digital examination was more difficult than the occiput anterior position. Ultrasound examination is an accurate method to determine foetal head position, but ultrasound equipment is expensive and not available worldwide. In economically underdeveloped areas, digital examination is still an economical and effective way to determine foetal head position. Our study investigated whether our additional new teaching methods could increase the accuracy of residents’ transvaginal digital examination of foetal head position.

Inappropriate estimation of the foetal head position is associated with prolonged labour, a higher rate of caesarean section, postpartum haemorrhage, and a higher rate of morbidity for the mother and the newborn [13, 14]. Compared with the occiput anterior position, the foetus in the occiput posterior or occipitotransverse position has higher rates of asphyxia neonatorum, neonatal intensive care unit (NICU) admissions, and longer hospitalizations [15]. Foetal head position is an important factor for predicting the progress of labour and determining the management of labour. To correct foetal position, the obstetrician must first determine the foetal head position. However, transvaginal digital examination has a higher error rate in determining foetal head positation [16]. The foetal head position is closely related to the duration of labour, vaginal midwifery placement of vacuum delivery system and forceps and perinatal outcome [14]. If the foetal head position is occipital posterior or occipital transverse by digital examination, we can change maternal postures to correct foetal head position [17, 18]. In our study, if the foetus was in the left occipitoposterior or occipitotransverse position, we directed the pregnant woman to lie in a left prone position, and if the foetus was in the right occipitoposterior or occipitotransverse position, we direct the pregnant woman to lie in a right prone position.

The skull of the foetal head is soft, with soft tissue oedema of the foetal head, overlapping of the skull, and deformation of the bone joints because of the compression of the birth canal during labour, which leads to difficulties in identifying the position of the foetal head for inexperienced residents. Our study showed that the accuracy of digital examination of the foetal head position was higher in resident with an additional theoretical training. The findings of this study suggest that residents did not have a deep understanding of the theoretical knowledge of examining foetal head position, which is an important reason for the high failure rate of determining the foetal head position. Our new theory simplifies the method of determining the anterior fontanelle and posterior fontanelle, allowing residents to acquire the skills to determine the position of the foetal head in a relatively short period of time. This kind of education can include theoretical teaching before entering clinical practice and then real-time teaching on the labour unit to increase the depth of understanding of foetal head position examination. Ultrasonography can help unexperienced residents improve the accuracy of digital vaginal examination in determining the position of the foetal head [19, 20]. However, we still believe that digital vaginal examination is an important way that obstetricians should have in the process of labour to evaluate the position of the foetal head, presence of caput succedaneum, presence of moulding and foetal head station. Especially when foetal heart rate deceleration or abnormal labour occurs, digital vaginal examination is the most rapid and economic examination means. Caesarean section rates remain high in some parts of our country, residents have fewer opportunities to perform forceps or vacuum delivery during training. Our additional theoretical training program can improve the accuracy of fetal head position diagnosis, help residents to deal with the labour process, and improve the success rate of vaginal delivery.

One of the strengths of our study is that the additional theoretical training we propose can significantly reduce the time residents need to be skilled in determining foetal head position through vaginal examination. Additionally, our theoretical training can improve the accuracy of vaginal examination of the foetal head position and facilitate vaginal delivery. This study has some limitations, ultrasound is the standard for diagnosing foetal head position, but it is not 100% accurate. The study was conducted in one hospital, so the sample size was relatively small. We need a larger sample size to provide evidence for these results.

## Conclusion

In conclusion, an additional theoretical training program can increase the accuracy of vaginal assessment of foetal head position for residents. After timely determination of foetal head position by transvaginal digital examination, we can correct the foetal head position through maternal posture change or MRFO, which can be helpful to manage labour dystocia and increase the rate of vaginal delivery. In the future, we can develop foetal skull suture models under normal conditions and models when skulls overlap during labour for the training of residents. In addition to doctors, midwives and nurses can also benefit from this training intervention.

## Data Availability

All data generated or analysed during this study are included in this published article.

## Abbreviations

MRFO: Manual rotation of the foetal occiput
SD: Standard deviation
NICU: Neonatal intensive care unit
